# Assessing the dimensionality of the EQ-HWB-25 alongside EQ-5D-5L, QOL-ACC and ASCOT in an older adult population

**DOI:** 10.1007/s11136-026-04315-8

**Published:** 2026-06-22

**Authors:** Mina Bahrampour, Akanksha Akanksha, Maja Kuharic, Rosalie Viney, Brendan Mulhern

**Affiliations:** 1 Centre for Health Economics Research and Evaluation, University of Technology Sydney, Sydney, NSW Australia; 2 Department of Medical Social Sciences, Northwestern University Feinberg School of Medicine, Chicago, IL USA

**Keywords:** Quality of life, Psychometrics, Factor analysis, Validity, EQ-HWB

## Abstract

**Purpose:**

Several instruments assess different aspects of quality of life (QoL), the EQ-HWB is developed to capture broader health and wellbeing constructs. Established QoL measures such as the EQ-5D-5L, QOL-ACC, and ASCOT are widely used in older populations. Analysing dimensionality across these instruments can provide insight into the constructs they cover and their conceptual relationships. This study aimed to examine the dimensionality of the EQ-HWB both on its own and alongside pooled items from these measures, to identify its underlying structure and the extent of item overlap.

**Methods:**

Analysis was conducted using data from 453 participants aged 65 years and above who completed all four instruments. Exploratory Factor Analysis (EFA) was conducted to identify the underlying factor structure. Factors were selected based on eigenvalues greater than one and scree plots. A correlation cut-off of 0.32 was applied to determine item loading on a given factor. Both oblique and orthogonal rotations were explored. EFA was conducted separately for the EQ-HWB and each instrument, as well as for the pooled items from all instruments.

**Results:**

EFAs conducted separately for the EQ-HWB, and each instrument resulted in a 4-factor structure. However, EFA of all pooled items showed that the 5-factor structure provided a better model fit. The five factors identified were: emotional functioning, self-care and usual activities, leisure and enjoyment, cognition and senses, and pain.

**Conclusion:**

This study provides evidence on the dimensions of QoL captured by a select pool of health focused and broader QoL instruments in an older adult population. The results enhance understanding of the conceptual coverage of the EQ-HWB relative to other QoL measures.

**Supplementary Information:**

The online version contains supplementary material available at https://doi.org/10.1007/s11136-026-04315-8.

## Simple English Summary

This study is done as there are many instruments measuring quality of life (QoL) in older adults, but it is not always clear how similar or different they are. The EQ-HWB is a newer measure designed to capture both health and broader wellbeing. However, more evidence is required to understand what areas it measures and how it compares with established tools such as the EQ-5D-5L, QOL-ACC, and ASCOT. The key issue addressed in this manuscript is whether the EQ-HWB measures the same dimensions of QoL as other instruments, and how much overlap exists between them. Understanding this helps researchers and policymakers choose the most appropriate measure. The main aim of the study was to examine the underlying structure of the EQ-HWB on its own and when combined with items from other QoL instruments. The results showed that when analysed separately, four main factors were seen. However, when all items were analysed together, five broader factors were identified: emotional functioning, self-care and usual activities, leisure and enjoyment, cognition and senses, and pain. These findings suggest that the EQ-HWB captures key areas of QoL and shares some overlap with existing measures, while also covering broader aspects of wellbeing.

**Supplementary Information:**

The online version contains supplementary material available at https://doi.org/10.1007/s11136-026-04315-8.

## Introduction

Various instruments have been developed to measure different aspects of quality of life (QoL), with each reflecting a particular concept and policy framework. The EQ-5D-5L is widely used in health economics to capture health-related quality of life (HRQoL) [1], whereas EQ Health and Wellbeing instrument (EQ-HWB) measures were developed to capture a broader concept that extends beyond health to include domains relevant health and social care, reflecting impacts on patients, social care users, and unpaid carers. [2]. The EQ-HWB has an experimental 25-item and nine item versions available intended to use for patients, social care users and carers, and enables the estimation of quality adjusted life years (QALYs) for interventions across different settings such as health and social care. This broader scope potentially makes the EQ-HWB suitable for cross-sector comparisons, particularly in contexts where outcomes from social care interventions need to be compared with those from healthcare, but its properties and applicability across sectors require further empirical exploration [3, 4].

Other instruments, such as the Quality of Life-Aged Care Consumers (QOL-ACC) [5] and the Adult Social Care Outcomes Toolkit (ASCOT) [6], are explicitly designed for use in aged care and social care settings, respectively. These tools capture dimensions such as autonomy, control over daily life and social participation, domains that are not typically included in health-related measures due to their different developmental frameworks. Each instrument is grounded in a specific theoretical and policy context, but the extent to which these theoretical structures are supported by empirical evidence has varied across studies. Therefore, assessing the underlying dimensionality is important for understanding the domains they measure. Dimensionality assessment examines the underlying constructs captured by different instruments and can provide a better understanding of the relationships between various QoL measures.

Prior studies have examined the conceptual overlap between QoL measuress and their suitability for different evaluative contexts, with much of this work initially motivated by the development of EQ-5D bolt-on dimensions to determine whether additional items captured latent constructs not represented in core health measures [7]. These early pooled-item analyses established a methodological framework for evaluating shared and distinct latent structures across instruments but were largely limited to established measures such as the EQ-5D and ASCOT [8, 9]. More recently the dimensionality of the EQ-HWB has been assessed in recent studies, typically alongside the EQ-5D and across different populations and conditions [10, 11]; however, evidence remains limited on how EQ-HWB items perform when analysed jointly with other contemporary QoL measures. Conducting Exploratory Factor Analysis (EFA) on pooled items provides a rigorous approach to clarifying conceptual overlap and distinctiveness across instruments, addressing a key gap in the psychometric evidence base [12].

Measuring QoL in older populations presents specific challenges due to the complex nature of ageing-related decline and the increasing importance of non-health factors, such as independence, security, and social connectedness [13, 14]. Given the growing demand for integrated care and cross-sector resource allocation [15], there is a need for instruments that can measure QoL comprehensively and consistently across settings. Dimensionality assessment can help determine whether the instruments included in a study capture the domains most relevant to older populations. In addition, it highlights the relationships between different measures, supporting more comprehensive and meaningful QoL assessment.

Assessing the dimensionality of the EQ-HWB-25 (hereafter referred to as EQ-HWB) alone, and alongside items from ASCOT, QOL-ACC, and EQ-5D-5L will provide stronger evidence of its construct validity and its alignment with, or divergence from, other QoL instruments [16]. This study aims to assess the dimensionality of the EQ-HWB to investigate its underlying structure and to examine the dimensionality of pooled items from the EQ-HWB and other QoL measures (EQ-5D-5L, QOL-ACC, and ASCOT) to evaluate the extent of overlap and their conceptual relationships. [17]. This work contributes to the emerging evidence base on the measurement properties of the EQ-HWB.

## Methods

### Data

The data was obtained from a 2023 online longitudinal study in Australia that collected self-reported health conditions and QoL data. Data collection was carried out in Australia between May and July 2023. The survey recruited a population sample through the panel company called ‘Pureprofile’. A stratified sampling approach was used to ensure representation of the Australian adult population by gender, age group, and state or territory of residence, in accordance with the 2021 Australian Census. Details regarding study data can be found in another paper [18]. The analysis in this paper focuses on a subset of 453 Australians (aged 65 and older) who completed the survey and answered the EQ-5D-5L, EQ-HWB, QOL-ACC, and ASCOT questionnaires. The ASCOT and EQ-5D-5L surveys were randomly assigned to participants while the QOL-ACC and EQ-HWB were fixed and QOL-ACC was just presented to participants who were 65 + age. Details about each instrument can be found in Table 1.

**Table 1:** Instrument Details

Instrument Name	domains/	Number of levels/levels	Domains	Recall Period	Focus
EQ-HWB [19]	7 domains (experimental 25 items)	5 levels (frequency, severity, or difficulty scales.)	Activity, Autonomy, Cognition, Feelings and Emotions, Relationships, Physical Sensations, And Self-Identity	Last 7 days	Health, Social care and Carer-related QoL
EQ-5D-5L [20]	5 domains with	5 levels (severity scale)	Mobility, Self-Care, Usual Activities, Pain|Discomfort, Anxiety|Depression	Today	HRQoL
ASCOT [21]	8 domains (9 items)	4 levels (severity, or intensity of the need scale)	Control over daily life, Personal cleanliness and comfort, Food and drink, Personal safety, Social participation and involvement, Occupation, Accommodation Cleanliness and comfort, Dignity	-	Social care related Qol (ScRQoL)
QOL-ACC [22]	6 domains	5 levels (frequency scale)	Independence, Mobility, Emotional Wellbeing, Social Connections, Activities, Pain Management	Today	QoL in aged care

### Assessment of convergent validity

Correlation among observed variables was used in the factor analysis to identify underlying latent constructs. We examined the distribution of item responses to determine the appropriate correlation method. The distribution of item responses was examined to determine the appropriate correlation method. To support the assessment of dimensionality, convergent validity was examined through Spearman’s rank correlations, appropriate for non-normally distributed data. Convergent validity indicates the extent to which the domains and items included in instruments measure overlapping constructs. Correlation coefficients were interpreted using predefined thresholds, values < 0.3 were considered weak,between 0.3 to < 0.5 moderate, and ≥ 0.5 strong [23]. These analyses were conducted to determine the extent to which the instruments measure similar constructs, and which will not. It is hypothesised that at least items that measure the same underlying construct have a strong correlation. For instance, items related to emotional functioning such as emotional wellbeing, anxiety, sadness depression are expected to be highly correlated or items related to pain which are in all instruments except ASCOT are expected to be highly correlated.

### Exploring dimensionality using factor analysis

Exploratory factor analysis was employed to identify and measure underlying latent constructs that are not directly observable. Given the objective of examining the dimensional structure of the item pool without applying a predefined model, EFA was an appropriate method for this analysis. A total of 44 items were pooled from the instruments, and the sample size was 453, giving an item-to-response ratio of approximately 1:10. This indicates an adequate sample size for conducting the EFA [24].

To assess the overall dimensionality, items from the four instruments were pooled. The Stata default EFA method, principal factor, was used for the estimation method. To evaluate the suitability of the dataset for EFA, the Kaiser–Meyer–Olkin (KMO) measure and Bartlett’s test of sphericity were conducted. The KMO statistic assesses sampling adequacy by measuring the proportion of shared variance among variables, with values between 0.8 and 1.0 indicating that the data are appropriate for factor analysis (FA) [25]. Bartlett’s test was used to confirm the presence of meaningful correlations among variables that justify using factor analysis. A *p*-value < 0.05 was considered evidence of sufficient inter-item correlation to proceed with EFA [26]. Mardia’s test for skewness was used to evaluate whether the data were multivariate normal [27].

The factor structure was explored using eigenvalues and scree plots. Usually, factors are chosen based on eigenvalues greater than 1. However, it is also possible to specify the number of factors to extract, a choice that can be supported by scree plots. Items were included in a factor if their loadings exceeded 0.32 [28]. In instances of cross-loading (i.e., loadings > 0.32 on more than one factor), the item was assigned to the factor with the highest loading. An oblique Promax rotation method was applied to account for potential correlations between factors [29].

To examine the underlying structure of the EQ-HWB and its relationship with other measures, EFA was conducted on: (1) the EQ-HWB independently, (2) EQ-HWB in combination with each instrument separately, and on (3) a pooled set of items from all instruments. For the relationship of EQ-HWB with each instrument we explored the EFA result from eigenvalues > 1 and scree plots.

Polychoric FA models were also tested. Due to high inter-item correlations between the EQ-5D and EQ-HWB, the resulting polychoric factor correlation matrix was non–positive definite, preventing factor extraction. To address this, several highly correlated items such as mobility and pain had to be excluded. However, given the objective of evaluating the dimensionality of all items in this study, the results presented are based on the complete item set using EFA.

All analyses were performed using Stata version 18.0 (StataCorp LLC, College Station, TX, USA).

### Ethics

The ethics for this study was obtained from the UTS Human Research Ethics Committee (CHERE program; HREC number ETH21-6090). Participants provided consent before participating in the main (baseline) and follow-up surveys.

## Results

### Sample

A total number of 1505 completed the survey of whom 453 participants met the inclusion criteria of being aged 65 and above and completing all four instruments. The mean age was 74.7 years (SD = 6.0), and 259 participants (57.17%) were male. Most of the participant (~ 66.66%) have reported good health (good, very good excellent). Table 2 provides a summary of the sample’s demographic characteristics.

**Table 2:** Summary of demographic characteristics

		N	(%)
Age(years)	65–69	108	23.84
	70–74	74	16.34
	75–79	188	41.50
	80–84	56	12.36
	85–90	24	5.30
	90–94	3	0.66
Gender	Male	259	57.17
	Female	194	42.83
Education	Year 12 or below	178	39.29
	Certificate or Diploma	167	36.87
	Bachelor’s degree	78	17.22
	Postgraduate	30	6.62
Employment	Retired	373	82.34
	Employed part-time	38	8.39
	Employed full-time	22	4.86
	Other	20	4.41
Self-report health status	Excellent	19	4.19
	Very good	94	20.75
	Good	189	41.72
	Fair	124	27.37
	Poor	27	5.96

### Convergent validity

The distribution of responses to the items in the ASCOT, EQ-5D-5L, QOL-ACC and EQ-HWB are shown in supplementary file (Figs. 1 s-4 s). The distribution of responses across levels was uneven for all instruments, with most participants selecting the first two response options, except for the positively worded EQ-HWB items (Feel good about yourself, doing thing you want to do, getting accepted by others) which the last two response levels were chosen the most. Tables for correlation between items can be found in appendix 1. There was only one item that showed a strong, although negative, correlation between the EQ‑HWB and ASCOT, the EQ‑HWB item “things you want to do” and the ASCOT “occupation” domain. The second highest correlation was between EQ-HWB item “feel you had control over day-to-day life” and ASCOT’s “control you have over your daily life” (~ 0.50).

There were strong correlations between items from EQ-HWB and QOL-ACC, all items from QOL-ACC had high correlations with at least one of the items from EQ-HWB except item “social relationships” which did not have strong correlation with any of the EQ-HWB items. The highest correlation was between “emotional wellbeing” from QOL-ACC and EQ-HWB item “had nothing to look forward to” (0.64).

All items from EQ-5D-5L had strong correlations with at least one item from EQ-HWB. The strongest correlation was between EQ-5D-5L’s “pain/ discomfort” and EQ-HWB’s “have physical pain” item (0.80). A study by the project team, in preparation, will give more details regarding the psychometric performance in this population.

### Factor analysis

***EQ-HWB****:* The KMO for the EQ-HWB data was 0.92 and bartlett test was significant. Eigenvalues higher than one showed a 3-factor structure accounting for 89.51% of the variance. A 4-factor structure was also tested as the eigenvalue for the fourth factor was almost one (0.97) and accounting for 96.29% of the variance. In the 4 factor structure items related to senses and cognition and safety loaded on the same factor. Items regarding pain except for “physical discomfort”, loaded on a separate factor. Items related to activities, such as mobility, selfcare and activities and the positively worded item “doing thing you wanted to do” load on another factor.^1^ While the remaining items mostly reflecting emotional functioning loaded on the final factor. Results have been shown in supplementary file appendix 1.

### EQ-HWB with each instrument

***EQ-HWB and ASCOT****:* The KMO for data from ASCOT and EQ-HWB was 0.92 and the Bartlett test was significant.^2^ Eigenvalues and scree plot suggested a 4-factor structure which would account for 87.93% of the total variance. Items from ASCOT loaded on 2 factors and EQ-HWB had items loading on all four factors. ASCOT “food and drink”, “accommodation” and “dignity” domains did not load on any factors. EQ-HWB items “difficulty hearing” and “physical discomfort” did not load on any of the factors. The unloaded items serve as single-item indicators which may capture unique aspects of the constructs not shared with other items. Therefore, they are still contributing information about specific domains or items that are not represented by the factors derived from the pooled items.

Factor 1 has one item from ASCOT related to social participation and 12 items from EQ-HWB items (which were related to exhaustion, loneliness, support also emotional functioning). The second factor has 4 items from ASCOT (related to cleanliness and control) and 4 items from EQ-HWB (related to activities and selfcare) which are mostly related to independence. Factors 3 and 4 only have items from EQ-HWB. The items loaded on factor 3 are all related to pain. And items loaded on factor 4 are seeing, cognition and safety related items.

***EQ-HWB and QOL-ACC****:* A KMO of 0.935 was observed for data from these two instruments with a significant Bartlett test. Eigenvalues and scree plot indicated a 4-factor structure accounting for 93.79% of the total variance. EQ-HWB items “difficulty hearing” and “sleep” and “physical discomfort” did not load on any of the factors. Items from QOL-ACC load on 3 factors. Factor 4 had EQ-HWB items regarding seeing, exhaustion, frustration, and cognition.

***EQ-HWB and EQ-5D-5L****:* Data from the EQ-HWB and EQ-5D-5L showed a KMO of 0.927 and Bartlett test was significant. A 4-factor structure was suggested by the results of the scree plot and Eigenvalues which account for 93.11% of the total variance. EQ-5D items loaded on the factors however, EQ-HWB items “difficulty hearing” and “physical discomfort” did not load on any factor. Items from EQ-5D loaded on three of the 4 factors. Factor 4 had items regarding seeing and cognition from EQ-HWB.

***All items pooled****:* Items pooled from all instruments showed a KMO of 0.939 and Bartlett test was significant. Eigenvalues and scree plot suggested a 4-factor structure accounting for 86.45% of the total variance. However, for this part of the analysis, we also tested a five-factor structure because the eigenvalue exceeded 0.7 [30]. The five-factor structure, accounting for 89.41% of the variance, provided a more meaningful underlying construct for the items. Mardia’s test for skewness showed that the data were not multivariate normal, so principal factor was used for the factor extraction.

The five factors identified represent key domains, these domains were defined by a subset of authors based on an assessment of the items included. Factor 1 as emotional functioning with 14 items of which one item was from ASCOT, one item from EQ-5D-5L, two items from QOL-ACC and the rest from EQ-HWB. Factor 2 as self-care and usual activities with 9 items, this includes two items from ASCOT, 3 items from EQ-5D-5L, one from QOL-ACC and three from EQ-HWB. Factor 3 as leisure and enjoyment with 5 items, one from ASCOT, one from QOL-ACC and three from EQ-HWB. Factor 4 as cognition and senses with 4 items, all from ASCOT and factor 5 as pain with 5 items which includes 1 from EQ-5D-5L, 1 from QOL-ACC and 3 from EQ-HWB.

Items from EQ-HWB loaded on all 5 factors while EQ-5D-5L and ASCOT items only loaded on 3 factors and, QOL-ACC items loaded on 4. ASCOT “food and drink”, “Accommodation”, “Safety” and “Dignity” domains, QOL-ACC “mobility” and, EQ-HWB items “difficulty hearing” and “physical discomfort” did not reach the 0.32 threshold, therefore did not load on any of the factors. These items showed high uniqueness values (greater than 0.6), indicating that a substantial portion of their variability was not explained by the extracted factors. This may be due to weak correlations with other variables or issues with how the items were measured. “Dignity” from ASCOT has a uniqueness of 0.92 which shows the item is unique compared to other items in the analysis. The ASCOT domain “Social participant and involvement” QOL-ACC domains “Emotional wellbeing” and “Social relationships” all have cross loading on factors 1 and 3. EQ-HWB item “Having no control over day-to-day life” has cross loading on factors 1 and 2.

Three items from EQ-HWB have negative loadings, which shows the items negative correlation with other items on the factor. Table 3 presents the EFA of the ASCOT, EQ-5D-5L, QOL-ACC and EQ-HWB items.

**Table 3:** EFA results of pooled items

Measure	Item*	Factor 1	Factor 2	Factor 3	Factor 4	Factor 5	Uniqueness
EQ-HWB-25	Unsafe	0.39					0.49
EQ-HWB-25	Exhausted	0.39					0.48
EQ-HWB-25	Control	0.44					0.40
ASCOT	Social participant and involvement	0.50					0.46
QOL-ACC	Social relationships	0.53					0.48
QOL-ACC	Emotional wellbeing	0.57					0.33
EQ-HWB-25	Cope	0.59					0.36
EQ-HWB-25	Frustrated	0.67					0.37
EQ-HWB-25	No support	0.72					0.49
EQ-HWB-25	Anxious	0.78					0.30
EQ-HWB-25	Nothing to look forward	0.79					0.27
EQ-HWB-25	Lonely	0.83					0.40
EQ-5D	Anxiety	0.85					0.34
EQ-HWB-25	Sad or depressed	0.96					0.19
ASCOT	Control		0.32				0.56
ASCOT	Cleanliness		0.36				0.64
QOL-ACC	Independence,		0.47				0.44
EQ-HWB-25	Activities		0.72				0.29
EQ-HWB-25	Mobility		0.83				0.34
EQ-HWB-25	Selfcare		0.83				0.40
EQ-5D	Usual activities		0.84				0.29
EQ-5D	Mobility		0.88				0.29
EQ-5D	Selfcare		0.88				0.42
EQ-HWB-25	Feel good yourself			−0.71			0.32
EQ-HWB-25	Thing you want to do			−0.70			0.33
EQ-HWB-25	Accepted by others			−0.64			0.57
ASCOT	Occupation			0.36			0.47
QOL-ACC	Leisure activities/hobbies)			0.43			0.52
EQ-HWB-25	Hearing				0.32		0.87
EQ-HWB-25	Seeing				0.50		0.74
EQ-HWB-25	Trouble concentrating				0.73		0.39
EQ-HWB-25	Trouble remembering				0.73		0.52
QOL-ACC	Pain management					0.57	0.49
EQ-5D	Pain					0.76	0.27
EQ-HWB-25	Amount physical discomfort					0.76	0.76
EQ-HWB-25	Amount physical pain					0.90	0.90
EQ-HWB-25	Physical pain					0.90	0.21
EQ-HWB-25	Discomfort						0.72
ASCOT	Food and drink						0.74
ASCOT	Safety						0.69
ASCOT	Accommodation						0.79
ASCOT	Dignity						0.92
QOL-ACC	Mobility						0.76
EQ-HWB-25	Sleep						0.68

^*^Item definition can be found in the appendix

## Discussion

In this study, we assessed the convergent validity and dimensionality of the EQ-HWB alongside three established HRQoL, ScRQoL and QoL instruments, EQ-5D-5L, ASCOT, and QOL-ACC respectively, using data collected from a sample of older Australians. Using EFA on EQ-HWB items and pooled item sets, we investigated how the EQ‑HWB’s domain structure overlaps with the other measures.

Our findings indicate that the EQ-HWB captures a broad concept of QoL, consistent with its design as a generic, cross-sectoral measure. Specifically, EQ‑HWB items loaded across all five factors identified in the pooled EFA—emotional functioning, self‑care and usual activities, leisure and enjoyment, cognition and senses, and pain—whereas EQ‑5D‑5L and ASCOT loaded on only three factors and QOL‑ACC on four. This finding aligns with prior research by Zhang et al. [11], which reported that EQ-HWB accounted for two additional factors—cognition and physical activity—not captured by the EQ-5D-5L. This highlights the multidimensional coverage of EQ-HWB and supports its use in settings that require comprehensive measurement across both health and non-health domains. The EFA of the EQ-HWB alone identified a four-factor structure, with one factor comprising exclusively positively worded items. This finding aligns with previous Australian studies [10, 31, 32], as well as research conducted in other countries [11, 17, 33] where the same three positively worded items consistently loaded onto a separate factor. This pattern likely reflects a method effect associated with item wording, rather than substantive conceptual differences, and highlights the need to consider the influence of positive versus negative phrasing in the interpretation of factor structures.

Cognition-related items from the EQ-HWB consistently formed distinct factors and did not co-load with items from the other instruments. This suggests that cognition represents a unique and meaningful construct not captured by ASCOT, QOL-ACC, or EQ-5D-5L. The importance of cognitive function in older adults' QoL has been well-documented and its absence from many generic measures represents a significant gap [34]. This supports its inclusion either as a bolt-on (as is being pursued for the EQ-5D-5L) or as part of broader instruments such as EQ-HWB. Similarly, the EQ-HWB item related to vision (“seeing”) tended to load with cognition, indicating a potential shared latent construct encompassing cognitive and sensory domains in older population [35–37]. As neither vision nor cognition are part of the core EQ-5D-5L but have been proposed as bolt-ons, our findings reinforce that these domains are not directly or indirectly assessed in other QoL measurement tools [38].

Despite some convergence across instruments in domains such as pain, self-care, and emotional wellbeing, some items exhibited high uniqueness or failed to load on any factor. These included ASCOT domains such as “dignity,” “food and drink,” and “safety,” as well as QOL-ACC “mobility” and EQ-HWB “difficulty hearing” and “physical discomfort.” High uniqueness can show that these items are not well explained by the common factor structure, suggesting that their variance is largely item specific or unrelated to the shared latent factors identified in the analysis. This may reflect context-specific content, method variance (e.g., distinctive wording, polarity, or recall period), or low prevalence in this sample. Either way, such items contribute little to the common factor solution, raising questions about their cross-sector generalisability. Follow up work should examine item level fit in an IRT framework.

There were also instances of cross-loadings, such as ASCOT’s “social participation” and QOL-ACC’s “emotional wellbeing” items, which loaded on both emotional and leisure/enjoyment-related factors. Similarly, the EQ-HWB item “having no control over day-to-day life” cross-loaded on both emotional and functional factors. These patterns may reflect the inherently multidimensional nature of these constructs rather than measurement error and suggest complex interdependencies between domains such as autonomy, mood, and social connectedness. The EQ-HWB item “feeling unsafe” loaded moderately (~ 0.39) on the emotional wellbeing factor, whereas ASCOT’s “feeling safe” did not load on any factor. This discrepancy may reflect differences in item wording (negative vs. positive) or recall periods, both of which can affect how respondents interpret and respond to questions.

Additionally, some positively worded EQ-HWB items (reverse worded) —such as “feeling accepted by others,” “feeling good about yourself,” and “doing things you want”—loaded negatively on the ‘leisure and enjoyment’ factor. These inverse loadings may reflect response polarity effects, scale directionality, or possible semantic misalignment with negatively framed items in the same factor. Further psychometric testing is warranted to understand the implications of item polarity on factor interpretation.

Another domain-specific observation involved the item “physical discomfort” from the EQ-HWB, which did not load with the pain-related items and showed weak correlations with the ‘pain’ factor. This may reflect the broader conceptualisation of discomfort in EQ-HWB (e.g., including symptoms like breathlessness or itchiness) compared to the narrower focus of “pain” in other instruments. This distinction has implications for how symptom burden is measured and valued, particularly in populations with multiple or atypical physical symptoms.

Finally, the findings have implications for instrument selection in older adult populations, given that domains such as cognition, sensory functioning, emotional wellbeing, and leisure featured prominently in the factor structure. Instruments like the EQ-HWB, which capture a broader range of these domains, may offer improved sensitivity and relevance in ageing-related QoL assessments, especially in cross-sectoral evaluations involving health, social care, and aged care.

Several limitations should be considered when interpreting these findings. First, we used EFA rather than confirmatory factor analysis (CFA), as our aim was exploratory rather than testing pre-specified models. Future research could use CFA to validate the factor structure identified here. Second, psychometric analyses such as EFA are sample-dependent and data driven, and results may differ in other populations or settings. It is important to note that the assessment is limited to the measures included in the design, and not all possible instruments can be incorporated, which imposes limits on the interpretation of the findings. Third, our sample was limited to Australians aged 65 and older who could complete an online survey, potentially excluding those with severe cognitive impairment or limited digital literacy. No post-stratification weighting or representativeness adjustments were applied as the data were from the general population study representative of age and gender; therefore, the findings should be interpreted with caution when generalising to the broader older population. Fourth, we focused on generic instruments and did not include condition-specific measures that might capture additional relevant domains. Finally, the cross-sectional nature of the data prevents assessment of temporal stability or responsiveness of the identified factor structure.

## Conclusion

This study provides empirical evidence that the EQ-HWB captures a broader range of domains than traditional health-focused measures in an older adult population. The comprehensive coverage across all identified factors supports its potential utility for cross-sectoral evaluations spanning health, social, and aged care settings. However, the high uniqueness of certain items and cross-loadings suggest areas for potential refinement. These findings can inform both the selection of appropriate instruments for older adult populations and the ongoing development of the EQ-HWB and other QoL measures.

## Supplementary Information

Below is the link to the electronic supplementary material.

Supplementary file1 (DOCX 205 KB)

## Data Availability

No datasets were generated or analysed during the current study.

## Footnotes

1 Result tables for EFA can be found in the appendix 1.

2
*p*-value 0.000.
