# Supplementary material for: Assessing the dimensionality of the EQ-HWB-25 alongside EQ-5D-5L, QOL-ACC and ASCOT in an older adult population
Source: Qual Life Res. 2026 Jun 22;35(8):205. doi: 10.1007/s11136-026-04315-8 (PMC13269440; doi:10.1007/s11136-026-04315-8)
Supplement: Supplementary file 1 — Supplementary file1 (DOCX 205 KB) [file 11136_2026_4315_MOESM1_ESM.docx]

**Assessing the Dimensionality of the EQ-HWB-25 Alongside EQ-5D-5L, QOL-ACC and ASCOT in an Older Adult Population**

**Authors:**

Mina Bahrampour ^1*^, Akanksha Akanksha ^1,^ Maja Kuharic ^2^, Rosalie Viney ^1^, Brendan Mulhern ^1^

^1^ Centre for Health Economics Research and Evaluation, University of Technology Sydney, NSW, Australia

^2^ Department of Medical Social Sciences, Northwestern University Feinberg School of Medicine, Chicago, Illinois, USA

*Corresponding author

Mina.bahrampour@uts.edu.au

*For Quality of life research journal*

**SUPPLEMENTARY FILE :**


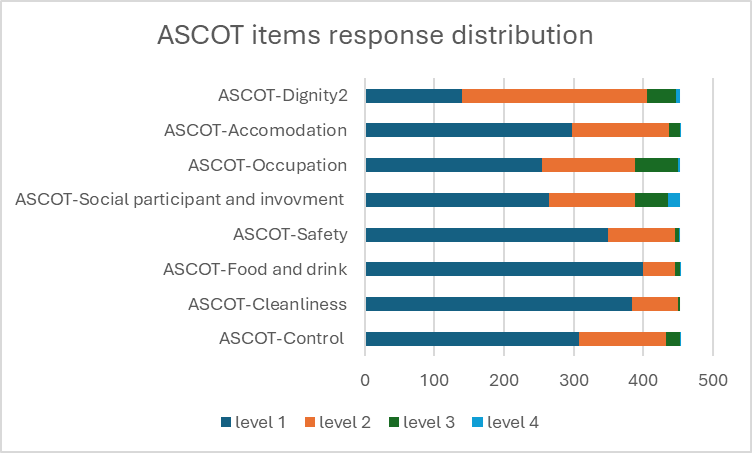


Figure 1s ASCOT items response distribution


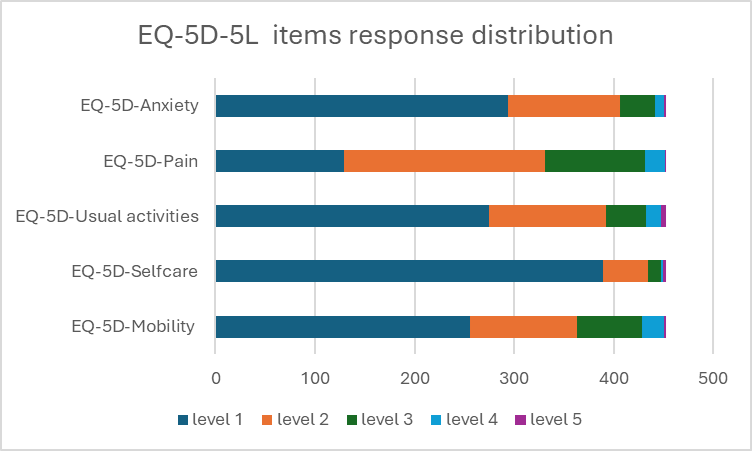


Figure 2s EQ-5D-5L items response distribution


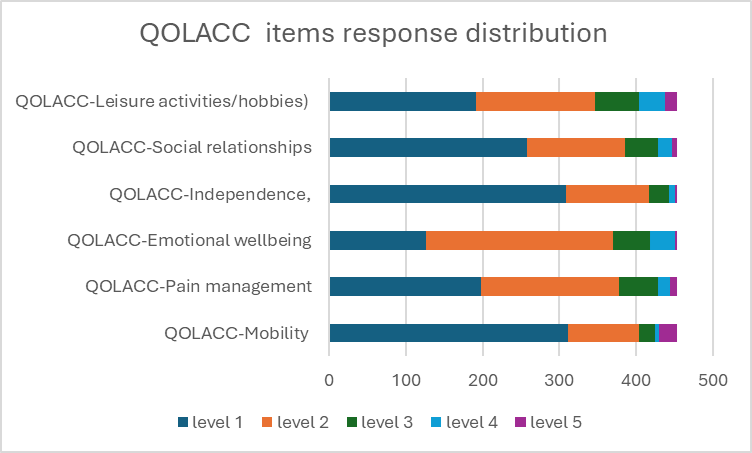


Figure 3s QOL-ACC items response distribution


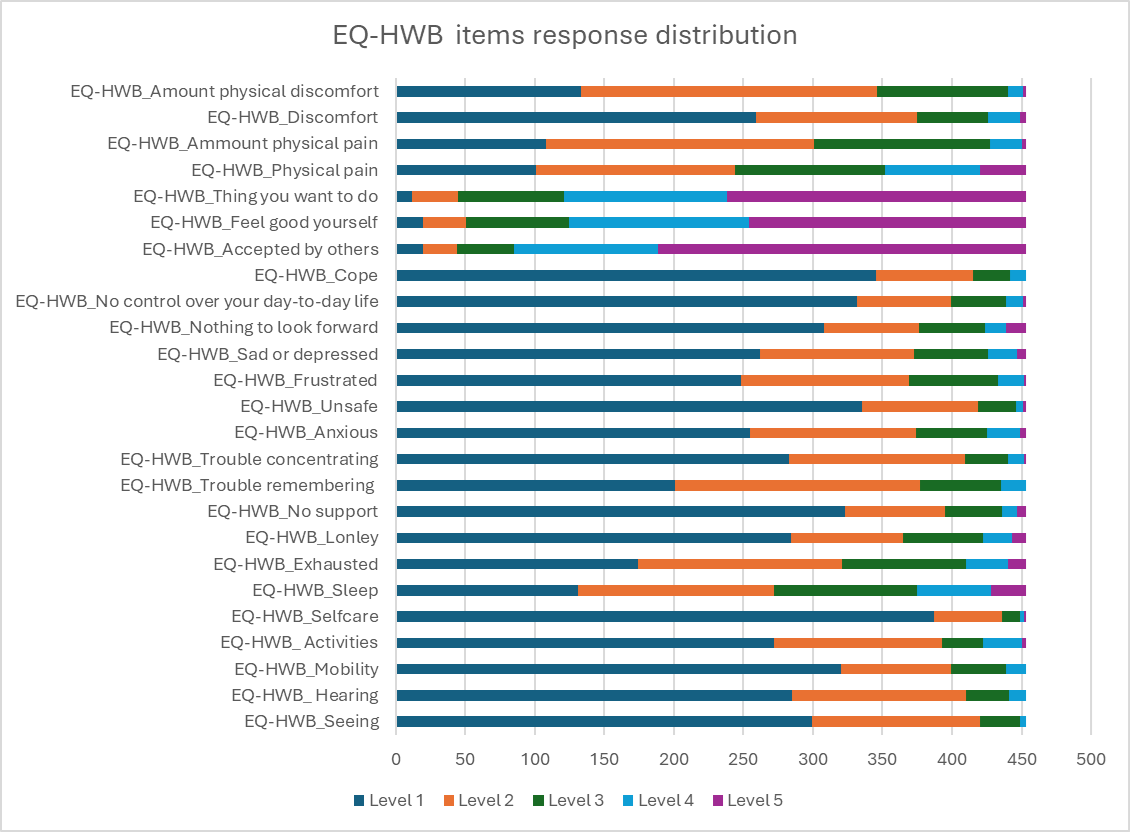


Figure 4s EQ-HWB items response distribution

**Appendix 1:**

**Correlation tables:**

Table 1a Correlation between EQ-HWB items and ASCOT items

|  | **ASCOT-CONTROL** | **ASCOT-CLEANLINESS** | **ASCOT-FOOD AND DRINK** | | **ASCOT-SAFETY** | **ASCOT-SOCIAL PARTICIPANT** | **ASCOT-OCCUPATION** | **ASCOT-ACCOMMODATION** | **ASCOT-DIGNITY** |
| --- | --- | --- | --- | --- | --- | --- | --- | --- | --- |
| **EQ-HWB- SEEING** | 0.26 | 0.18 | 0.16 | 0.25 | | 0.20 | 0.30 | 0.20 | 0.06 |
| **EQ-HWB-HEARING** | 0.22 | 0.12 | 0.14 | 0.17 | | 0.07 | 0.18 | 0.12 | 0.09 |
| **EQ-HWB-MOBILITY** | 0.33 | 0.30 | 0.30 | 0.29 | | 0.26 | 0.31 | 0.27 | 0.07 |
| **EQ-HWB-ACTIVITIES** | 0.42 | 0.34 | 0.32 | 0.29 | | 0.26 | 0.45 | 0.30 | 0.07 |
| **EQ-HWB-SELFCARE** | 0.37 | 0.38 | 0.29 | 0.27 | | 0.25 | 0.32 | 0.27 | 0.07 |
| **EQ-HWB-SLEEP** | 0.28 | 0.18 | 0.22 | 0.18 | | 0.25 | 0.32 | 0.25 | 0.09 |
| **EQ-HWB-EXHAUSTED** | 0.35 | 0.22 | 0.26 | 0.25 | | 0.28 | 0.44 | 0.23 | 0.11 |
| **EQ-HWB-LONELY** | 0.25 | 0.26 | 0.19 | 0.21 | | 0.46 | 0.36 | 0.30 | 0.15 |
| **EQ-HWB-NO SUPPORT** | 0.33 | 0.30 | 0.26 | 0.29 | | 0.43 | 0.40 | 0.29 | 0.15 |
| **EQ-HWB-TROUBLE REMEMBERING** | 0.30 | 0.24 | 0.17 | 0.21 | | 0.14 | 0.32 | 0.21 | 0.08 |
| **EQ-HWB-TROUBLE CONCENTRATING** | 0.31 | 0.27 | 0.15 | 0.24 | | 0.21 | 0.33 | 0.25 | 0.02 |
| **EQ-HWB-ANXIOUS** | 0.31 | 0.25 | 0.26 | 0.33 | | 0.37 | 0.40 | 0.27 | 0.09 |
| **EQ-HWB-UNSAFE** | 0.40 | 0.30 | 0.20 | 0.48 | | 0.26 | 0.38 | 0.25 | 0.07 |
| **EQ-HWB-FRUSTRATED** | 0.42 | 0.25 | 0.28 | 0.39 | | 0.39 | 0.48 | 0.28 | 0.18 |
| **EQ-HWB-SAD OR DEPRESSED** | 0.35 | 0.23 | 0.24 | 0.31 | | 0.45 | 0.43 | 0.26 | 0.17 |
| **EQ-HWB-NOTHING TO LOOK FORWARD** | 0.40 | 0.31 | 0.33 | 0.31 | | 0.47 | 0.47 | 0.31 | 0.18 |
| **EQ-HWB-NO CONTROL OVER YOUR DAY-TO-DAY LIFE** | 0.50 | 0.37 | 0.33 | 0.39 | | 0.39 | 0.48 | 0.31 | 0.10 |
| **EQ-HWB-COPE** | 0.39 | 0.36 | 0.35 | 0.40 | | 0.41 | 0.47 | 0.31 | 0.09 |
| **EQ-HWB-ACCEPTED BY OTHERS** | -0.27 | -0.30 | -0.25 | -0.19 | | -0.40 | -0.32 | -0.27 | -0.14 |
| **EQ-HWB- FEEL GOOD YOURSELF** | -0.41 | -0.37 | -0.33 | -0.26 | | -0.49 | -0.43 | -0.29 | -0.19 |
| **EQ-HWB-THING YOU WANT TO DO** | -0.46 | -0.38 | -0.35 | -0.30 | | -0.42 | -0.56 | -0.31 | -0.13 |
| **EQ-HWB-PHYSICAL PAIN** | 0.30 | 0.17 | 0.23 | 0.18 | | 0.19 | 0.29 | 0.23 | 0.05 |
| **EQ-HWB-AMOUNT PHYSICAL PAIN** | 0.32 | 0.20 | 0.24 | 0.25 | | 0.19 | 0.31 | 0.23 | 0.06 |
| **EQ-HWB-DISCOMFORT** | 0.30 | 0.30 | 0.22 | 0.26 | | 0.18 | 0.30 | 0.19 | 0.11 |
| **EQ-HWB-AMOUNT PHYSICAL DISCOMFORT** | 0.33 | 0.24 | 0.21 | 0.29 | | 0.21 | 0.31 | 0.25 | 0.01 |

Table 2a Correlation between EQ-HWB items and EQ-5D-5L items

|  | **EQ-5D-MOBILITY** | **EQ-5D-SELFCARE** | **EQ-5D-USUAL ACTIVITIES** | **EQ-5D-PAIN** | **EQ-5D-ANXIETY** |
| --- | --- | --- | --- | --- | --- |
| **EQ-HWB- SEEING** | 0.19 | 0.25 | 0.19 | 0.15 | 0.28 |
| **EQ-HWB-HEARING** | 0.11 | 0.16 | 0.10 | 0.15 | 0.12 |
| **EQ-HWB-MOBILITY-** | 0.76 | 0.53 | 0.64 | 0.48 | 0.22 |
| **EQ-HWB-ACTIVITIES** | 0.66 | 0.48 | 0.76 | 0.53 | 0.30 |
| **EQ-HWB-SELFCARE** | 0.50 | 0.69 | 0.53 | 0.41 | 0.29 |
| **EQ-HWB-SLEEP** | 0.14 | 0.18 | 0.18 | 0.33 | 0.33 |
| **EQ-HWB-EXHAUSTED** | 0.23 | 0.25 | 0.33 | 0.37 | 0.43 |
| **EQ-HWB-LONELY** | 0.17 | 0.20 | 0.24 | 0.25 | 0.55 |
| **EQ-HWB-NO SUPPORT** | 0.11 | 0.16 | 0.22 | 0.27 | 0.52 |
| **EQ-HWB-TROUBLE REMEMBERING** | 0.13 | 0.19 | 0.17 | 0.20 | 0.30 |
| **EQ-HWB-TROUBLE CONCENTRATING** | 0.08 | 0.23 | 0.20 | 0.22 | 0.44 |
| **EQ-HWB-ANXIOUS** | 0.02 | 0.15 | 0.17 | 0.19 | 0.73 |
| **EQ-HWB-UNSAFE** | 0.28 | 0.32 | 0.38 | 0.32 | 0.39 |
| **EQ-HWB-FRUSTRATED** | 0.23 | 0.27 | 0.34 | 0.33 | 0.61 |
| **EQ-HWB-SAD OR DEPRESSED** | 0.09 | 0.16 | 0.20 | 0.25 | 0.73 |
| **EQ-HWB-NOTHING TO LOOK FORWARD** | 0.19 | 0.23 | 0.27 | 0.25 | 0.59 |
| **EQ-HWB-NO CONTROL OVER YOUR DAY-TO-DAY LIFE** | 0.32 | 0.42 | 0.39 | 0.28 | 0.52 |
| **EQ-HWB-COPE** | 0.22 | 0.32 | 0.30 | 0.34 | 0.59 |
| **EQ-HWB-ACCEPTED BY OTHERS** | -0.15 | -0.19 | -0.16 | -0.14 | -0.36 |
| **EQ-HWB- FEEL GOOD YOURSELF** | -0.27 | -0.25 | -0.29 | -0.25 | -0.48 |
| **EQ-HWB-THING YOU WANT TO DO** | -0.36 | -0.33 | -0.45 | -0.35 | -0.43 |
| **EQ-HWB-PHYSICAL PAIN** | 0.47 | 0.27 | 0.42 | 0.80 | 0.22 |
| **EQ-HWB-AMOUNT PHYSICAL PAIN** | 0.49 | 0.29 | 0.45 | 0.79 | 0.25 |
| **EQ-HWB-DISCOMFORT** | 0.26 | 0.33 | 0.37 | 0.30 | 0.24 |
| **EQ-HWB-AMOUNT PHYSICAL DISCOMFORT** | 0.42 | 0.30 | 0.46 | 0.64 | 0.25 |

Table 3a Correlation between EQ-HWB items and QOL-ACC items

|  | **QOL-ACC-MOBILITY** | **QOL-ACC -PAIN MANAGEMENT** | **QOL-ACC -EMOTIONAL WELLBEING** | **QOL-ACC -INDEPENDENCE,** | **QOL-ACC -SOCIAL RELATIONSHIPS** | **QOL-ACC -LEISURE** |
| --- | --- | --- | --- | --- | --- | --- |
| **EQ-HWB- SEEING** | 0.25 | 0.22 | 0.23 | 0.30 | 0.21 | 0.20 |
| **EQ-HWB-HEARING** | 0.11 | 0.14 | 0.12 | 0.18 | 0.11 | 0.10 |
| **EQ-HWB-MOBILITY** | 0.57 | 0.34 | 0.23 | 0.42 | 0.19 | 0.23 |
| **EQ-HWB-ACTIVITIES** | 0.52 | 0.45 | 0.34 | 0.49 | 0.24 | 0.38 |
| **EQ-HWB-SELFCARE** | 0.47 | 0.37 | 0.28 | 0.43 | 0.25 | 0.25 |
| **EQ-HWB-SLEEP** | 0.21 | 0.37 | 0.40 | 0.30 | 0.26 | 0.30 |
| **EQ-HWB-EXHAUSTED** | 0.32 | 0.39 | 0.45 | 0.40 | 0.27 | 0.37 |
| **EQ-HWB-LONELY** | 0.23 | 0.34 | 0.50 | 0.33 | 0.42 | 0.45 |
| **EQ-HWB-NO SUPPORT** | 0.22 | 0.33 | 0.51 | 0.40 | 0.47 | 0.37 |
| **EQ-HWB-TROUBLE REMEMBERING** | 0.19 | 0.25 | 0.35 | 0.27 | 0.24 | 0.25 |
| **EQ-HWB-TROUBLE CONCENTRATING** | 0.18 | 0.28 | 0.38 | 0.31 | 0.26 | 0.28 |
| **EQ-HWB-ANXIOUS** | 0.16 | 0.36 | 0.54 | 0.36 | 0.41 | 0.38 |
| **EQ-HWB-UNSAFE** | 0.32 | 0.30 | 0.35 | 0.34 | 0.25 | 0.27 |
| **EQ-HWB-FRUSTRATED** | 0.31 | 0.37 | 0.58 | 0.47 | 0.43 | 0.42 |
| **EQ-HWB-SAD OR DEPRESSED** | 0.17 | 0.36 | 0.61 | 0.42 | 0.46 | 0.46 |
| **EQ-HWB-NOTHING TO LOOK FORWARD** | 0.26 | 0.36 | 0.64 | 0.38 | 0.48 | 0.51 |
| **EQ-HWB-NO CONTROL OVER YOUR DAY-TO-DAY LIFE** | 0.37 | 0.35 | 0.46 | 0.55 | 0.39 | 0.42 |
| **EQ-HWB-COPE** | 0.34 | 0.41 | 0.56 | 0.50 | 0.44 | 0.45 |
| **EQ-HWB-ACCEPTED BY OTHERS** | -0.27 | -0.24 | -0.45 | -0.32 | -0.43 | -0.39 |
| **EQ-HWB- FEEL GOOD YOURSELF** | -0.29 | -0.36 | -0.63 | -0.47 | -0.48 | -0.52 |
| **EQ-HWB-THING YOU WANT TO DO** | -0.42 | -0.44 | -0.55 | -0.57 | -0.42 | -0.52 |
| **EQ-HWB-PHYSICAL PAIN** | 0.36 | 0.62 | 0.30 | 0.33 | 0.23 | 0.22 |
| **EQ-HWB-AMOUNT PHYSICAL PAIN** | 0.37 | 0.59 | 0.30 | 0.35 | 0.18 | 0.24 |
| **EQ-HWB-DISCOMFORT** | 0.30 | 0.28 | 0.23 | 0.32 | 0.16 | 0.26 |
| **EQ-HWB-AMOUNT PHYSICAL DISCOMFORT** | 0.38 | 0.56 | 0.33 | 0.38 | 0.23 | 0.29 |

**EFAs:**

Table 4a EFA of EQ-HWB items (KMO=0.920)

| Variable | Factor1 | Factor2 | Factor3 | Factor4 | Uniqueness |
| --- | --- | --- | --- | --- | --- |
| **EQ-HWB- seeing** |  | 0.45 |  |  | 0.71 |
| **EQ-HWB- hearing** |  | 0.51 |  |  | 0.75 |
| **EQ-HWB-mobility** |  | 0.78 |  |  | 0.37 |
| **EQ-HWB- activities** |  | 0.70 |  |  | 0.30 |
| **EQ-HWB-selfcare** |  | 0.73 |  |  | 0.42 |
| **EQ-HWB-sleep** | 0.57 |  |  |  | 0.57 |
| **EQ-HWB-exhausted** | 0.71 |  |  |  | 0.42 |
| **EQ-HWB-lonely** | 0.74 |  |  |  | 0.41 |
| **EQ-HWB-no support** | 0.73 |  |  |  | 0.44 |
| **EQ-HWB-trouble remembering** | 0.67 |  |  |  | 0.57 |
| **EQ-HWB-trouble concentrating** | 0.85 |  |  |  | 0.39 |
| **EQ-HWB-anxious** | 0.87 |  |  |  | 0.30 |
| **EQ-HWB-unsafe** | 0.47 | 0.32 |  |  | 0.52 |
| **EQ-HWB-frustrated** | 0.79 |  |  |  | 0.36 |
| **EQ-HWB-sad or depressed** | 0.86 |  |  |  | 0.24 |
| **EQ-HWB-nothing to look forward** | 0.76 |  |  |  | 0.33 |
| **EQ-HWB-no control over your day-to-day life** | 0.64 |  |  |  | 0.40 |
| **EQ-HWB-cope** | 0.76 |  |  |  | 0.31 |
| **EQ-HWB-Accepted by others** |  |  |  | 0.73 | 0.40 |
| **EQ-HWB- feel good yourself** |  |  |  | 0.77 | 0.26 |
| **EQ-HWB-thing you want to do** |  |  |  | 0.74 | 0.32 |
| **EQ-HWB-physical pain** |  |  | 0.83 |  | 0.28 |
| **EQ-HWB-amount physical pain** |  |  | 0.86 |  | 0.23 |
| **EQ-HWB-discomfort** |  |  | 0.43 |  | 0.58 |
| **EQ-HWB-amount physical discomfort** |  |  | 0.76 |  | 0.32 |

Table 5a EFA between ASCOT and EQ-HWB (KMO=0.928)

| VARIABLE | Factor1 | Factor2 | Factor3 | Factor4 | Uniqueness |
| --- | --- | --- | --- | --- | --- |
| ASCOT-CONTROL |  | 0.53 |  |  | 0.57 |
| ASCOT-CLEANLINESS |  | 0.56 |  |  | 0.63 |
| ASCOT-FOOD AND DRINK |  |  |  |  | 0.74 |
| ASCOT-SAFETY |  | 0.37 |  |  | 0.67 |
| ASCOT-SOCIAL PARTICIPANT AND INVOLVEMENT | 0.61 |  |  |  | 0.53 |
| ASCOT-OCCUPATION | 0.36 | 0.38 |  |  | 0.50 |
| ASCOT-ACCOMMODATION |  |  |  |  | 0.80 |
| ASCOT-DIGNITY2 |  |  |  |  | 0.93 |
| EQ-HWB- SEEING |  |  |  | 0.41 | 0.75 |
| EQ-HWB-HEARING |  |  |  |  | 0.86 |
| EQ-HWB-MOBILITY- GETTING AROUND INSIDE AND OUTSIDE |  | 0.78 |  |  | 0.41 |
| EQ-HWB-ACTIVITIES |  | 0.77 |  |  | 0.31 |
| EQ-HWB-SELFCARE |  | 0.72 |  |  | 0.51 |
| EQ-HWB-SLEEP | 0.37 |  |  |  | 0.68 |
| EQ-HWB-EXHAUSTED | 0.42 |  |  |  | 0.46 |
| EQ-HWB-LONELY | 0.80 |  |  |  | 0.41 |
| EQ-HWB-NO SUPPORT | 0.72 |  |  |  | 0.50 |
| EQ-HWB-TROUBLE REMEMBERING |  |  |  | 0.56 | 0.58 |
| EQ-HWB-TROUBLE CONCENTRATING | 0.40 |  |  | 0.60 | 0.42 |
| EQ-HWB-ANXIOUS | 0.77 |  |  |  | 0.34 |
| EQ-HWB-UNSAFE |  |  |  | 0.42 | 0.49 |
| EQ-HWB-FRUSTRATED | 0.61 |  |  |  | 0.39 |
| EQ-HWB-SAD OR DEPRESSED | 0.88 |  |  |  | 0.24 |
| EQ-HWB-NOTHING TO LOOK FORWARD | 0.85 |  |  |  | 0.28 |
| EQ-HWB-NO CONTROL OVER YOUR DAY-TO-DAY LIFE | 0.38 | 0.35 |  |  | 0.42 |
| EQ-HWB-COPE | 0.61 |  |  |  | 0.37 |
| EQ-HWB-ACCEPTED BY OTHERS | -0.58 |  |  |  | 0.57 |
| EQ-HWB- FEEL GOOD YOURSELF | -0.65 | -0.34 |  |  | 0.34 |
| EQ-HWB-THING YOU WANT TO DO | -0.40 | -0.52 |  |  | 0.39 |
| EQ-HWB-PHYSICAL PAIN |  |  | 0.83 |  | 0.28 |
| EQ-HWB-AMOUNT PHYSICAL PAIN |  |  | 0.86 |  | 0.22 |
| EQ-HWB-DISCOMFORT |  |  |  |  | 0.72 |
| EQ-HWB-AMOUNT PHYSICAL DISCOMFORT |  |  | 0.75 |  | 0.33 |

Table 6a EFA between EQ-5D-5L and EQ-HWB (KMO=0.927)

| VARIABLE | Factor1 | Factor2 | Factor3 | Factor4 | Uniqueness |
| --- | --- | --- | --- | --- | --- |
| EQ-5D-MOBILITY |  | 0.83 |  |  | 0.29 |
| EQ-5D-SELFCARE |  | 0.84 |  |  | 0.43 |
| EQ-5D-USUAL ACTIVITIES |  | 0.83 |  |  | 0.28 |
| EQ-5D-PAIN |  |  | 0.75 |  | 0.27 |
| EQ-5D-ANXIETY | 0.78 |  |  |  | 0.38 |
| EQ-HWB- SEEING |  |  |  | 0.32 | 0.77 |
| EQ-HWB-HEARING |  |  |  |  | 0.88 |
| EQ-HWB-MOBILITY- GETTING AROUND INSIDE AND OUTSIDE | 0.79 |  |  |  | 0.33 |
| EQ-HWB-ACTIVITIES | 0.74 |  |  |  | 0.29 |
| EQ-HWB-SELFCARE | 0.78 |  |  |  | 0.41 |
| EQ-HWB-SLEEP | 0.35 |  |  |  | 0.68 |
| EQ-HWB-EXHAUSTED | 0.45 |  |  |  | 0.47 |
| EQ-HWB-LONELY | 0.75 |  |  |  | 0.46 |
| EQ-HWB-NO SUPPORT | 0.72 |  |  |  | 0.52 |
| EQ-HWB-TROUBLE REMEMBERING | 0.34 |  |  | 0.46 | 0.59 |
| EQ-HWB-TROUBLE CONCENTRATING | 0.48 |  |  | 0.51 | 0.40 |
| EQ-HWB-ANXIOUS | 0.85 |  |  |  | 0.30 |
| EQ-HWB-UNSAFE | 0.35 |  |  |  | 0.55 |
| EQ-HWB-FRUSTRATED | 0.70 |  |  |  | 0.38 |
| EQ-HWB-SAD OR DEPRESSED | 0.92 |  |  |  | 0.23 |
| EQ-HWB-NOTHING TO LOOK FORWARD | 0.89 |  |  |  | 0.29 |
| EQ-HWB-NO CONTROL OVER YOUR DAY-TO-DAY LIFE | 0.56 | 0.32 |  |  | 0.43 |
| EQ-HWB-COPE | 0.72 |  |  |  | 0.36 |
| EQ-HWB-ACCEPTED BY OTHERS | -0.66 |  |  | 0.42 | 0.55 |
| EQ-HWB- FEEL GOOD YOURSELF | -0.78 |  |  | 0.38 | 0.32 |
| EQ-HWB-THING YOU WANT TO DO | -0.58 | -0.34 |  | 0.33 | 0.40 |
| EQ-HWB-PHYSICAL PAIN |  |  | 0.87 |  | 0.22 |
| EQ-HWB-AMOUNT PHYSICAL PAIN |  |  | 0.89 |  | 0.19 |
| EQ-HWB-DISCOMFORT |  |  |  |  | 0.73 |
| EQ-HWB-AMOUNT PHYSICAL DISCOMFORT |  |  | 0.75 |  | 0.36 |

Table 7a EFA between QOL-ACC and EQ-HWB (KMO=0.935)

| VARIABLE | Factor1 | Factor2 | Factor3 | Factor4 | Uniqueness |
| --- | --- | --- | --- | --- | --- |
| QOL-ACC-MOBILITY |  | 0.42 |  |  | 0.75 |
| QOL-ACC -PAIN MANAGEMENT |  |  | 0.58 |  | 0.50 |
| QOL-ACC -EMOTIONAL WELLBEING | 0.80 |  |  |  | 0.32 |
| QOL-ACC -INDEPENDENCE, |  | 0.56 |  |  | 0.45 |
| QOL-ACC -SOCIAL RELATIONSHIPS | 0.70 |  |  |  | 0.55 |
| QOL-ACC -LEISURE (ACTIVITIES/HOBBIES) | 0.56 |  |  |  | 0.55 |
| EQ-HWB- SEEING |  |  |  | 0.42 | 0.76 |
| EQ-HWB-HEARING |  |  |  |  | 0.87 |
| EQ-HWB-MOBILITY- GETTING AROUND INSIDE AND OUTSIDE |  | 0.78 |  |  | 0.37 |
| EQ-HWB-ACTIVITIES |  | 0.75 |  |  | 0.30 |
| EQ-HWB-SELFCARE |  | 0.70 |  |  | 0.50 |
| EQ-HWB-SLEEP |  |  |  |  | 0.67 |
| EQ-HWB-EXHAUSTED |  |  |  | 0.35 | 0.47 |
| EQ-HWB-LONELY | 0.70 |  |  |  | 0.44 |
| EQ-HWB-NO SUPPORT | 0.65 |  |  |  | 0.50 |
| EQ-HWB-TROUBLE REMEMBERING |  |  |  | 0.58 | 0.58 |
| EQ-HWB-TROUBLE CONCENTRATING |  |  |  | 0.67 | 0.41 |
| EQ-HWB-ANXIOUS | 0.67 |  |  | 0.39 | 0.33 |
| EQ-HWB-UNSAFE |  |  |  | 0.42 | 0.54 |
| EQ-HWB-FRUSTRATED | 0.54 |  |  |  | 0.38 |
| EQ-HWB-SAD OR DEPRESSED | 0.78 |  |  |  | 0.25 |
| EQ-HWB-NOTHING TO LOOK FORWARD | 0.82 |  |  |  | 0.26 |
| EQ-HWB-NO CONTROL OVER YOUR DAY-TO-DAY LIFE | 0.37 | 0.36 |  |  | 0.41 |
| EQ-HWB-COPE | 0.57 |  |  |  | 0.37 |
| EQ-HWB-ACCEPTED BY OTHERS | -0.67 |  |  |  | 0.56 |
| EQ-HWB- FEEL GOOD YOURSELF | -0.76 |  |  |  | 0.31 |
| EQ-HWB-THING YOU WANT TO DO | -0.52 | -0.50 |  |  | 0.35 |
| EQ-HWB-PHYSICAL PAIN |  |  | 0.85 |  | 0.27 |
| EQ-HWB-AMOUNT PHYSICAL PAIN |  |  | 0.89 |  | 0.22 |
| EQ-HWB-DISCOMFORT |  |  |  |  | 0.72 |
| EQ-HWB-AMOUNT PHYSICAL DISCOMFORT |  |  | 0.79 |  | 0.33 |

**Correlation matrix of common factors for deciding on the rotation model:**

Table 8a Correlation matrix of common factors for deciding on the rotation model

Table 9a EFA of pooled items based on measures

| Measure | Item | factor 1 | factor 2 | factor 3 | factor 4 | factor 5 | Uniqueness |
| --- | --- | --- | --- | --- | --- | --- | --- |
| EQ-HWB | Unsafe | 0.39 |  |  |  |  | 0.49 |
| EQ-HWB | Exhausted | 0.39 |  |  |  |  | 0.48 |
| EQ-HWB | Control | 0.44 |  |  |  |  | 0.40 |
| ASCOT | Social participant and involvement | 0.50 |  |  |  |  | 0.46 |
| QOLACC | Social relationships | 0.53 |  |  |  |  | 0.48 |
| QOLACC | Emotional wellbeing | 0.57 |  |  |  |  | 0.33 |
| EQ-HWB | Cope | 0.59 |  |  |  |  | 0.36 |
| EQ-HWB | Frustrated | 0.67 |  |  |  |  | 0.37 |
| EQ-HWB | No support | 0.72 |  |  |  |  | 0.49 |
| EQ-HWB | Anxious | 0.78 |  |  |  |  | 0.30 |
| EQ-HWB | Nothing to look forward | 0.79 |  |  |  |  | 0.27 |
| EQ-HWB | Lonely | 0.83 |  |  |  |  | 0.40 |
| EQ-5D | Anxiety | 0.85 |  |  |  |  | 0.34 |
| EQ-HWB | Sad or depressed | 0.96 |  |  |  |  | 0.19 |
| ASCOT | Control |  | 0.32 |  |  |  | 0.56 |
| ASCOT | Cleanliness |  | 0.36 |  |  |  | 0.64 |
| QOLACC | Independence, |  | 0.47 |  |  |  | 0.44 |
| EQ-HWB | Activities |  | 0.72 |  |  |  | 0.29 |
| EQ-HWB | Mobility |  | 0.83 |  |  |  | 0.34 |
| EQ-HWB | Selfcare |  | 0.83 |  |  |  | 0.40 |
| EQ-5D | Usual activities |  | 0.84 |  |  |  | 0.29 |
| EQ-5D | Mobility |  | 0.88 |  |  |  | 0.29 |
| EQ-5D | Selfcare |  | 0.88 |  |  |  | 0.42 |
| EQ-HWB | Feel good yourself |  |  | -0.71 |  |  | 0.32 |
| EQ-HWB | Thing you want to do |  |  | -0.70 |  |  | 0.33 |
| EQ-HWB | Accepted by others |  |  | -0.64 |  |  | 0.57 |
| ASCOT | Occupation |  |  | 0.36 |  |  | 0.47 |
| QOLACC | Leisure activities/hobbies) |  |  | 0.43 |  |  | 0.52 |
| EQ-HWB | Hearing |  |  |  | 0.32 |  | 0.87 |
| EQ-HWB | Seeing |  |  |  | 0.50 |  | 0.74 |
| EQ-HWB | Trouble concentrating |  |  |  | 0.73 |  | 0.39 |
| EQ-HWB | Trouble remembering |  |  |  | 0.73 |  | 0.52 |
| QOLACC | Pain management |  |  |  |  | 0.57 | 0.49 |
| EQ-5D | Pain |  |  |  |  | 0.76 | 0.27 |
| EQ-HWB | Amount physical discomfort | |  |  |  |  | 0.76 |
| EQ-HWB | Amount physical pain | |  |  |  |  | 0.90 |
| EQ-HWB | Physical pain |  |  |  |  | 0.90 | 0.21 |
| EQ-HWB | Discomfort |  |  |  |  |  | 0.72 |
| ASCOT | Food and drink |  |  |  |  |  | 0.74 |
| ASCOT | Safety |  |  |  |  |  | 0.69 |
| ASCOT | Accommodation |  |  |  |  |  | 0.79 |
| ASCOT | Dignity |  |  |  |  |  | 0.92 |
| QOLACC | Mobility |  |  |  |  |  | 0.76 |
| EQ-HWB | Sleep |  |  |  |  |  | 0.68 |

Table 10a Abbreviation item definition

| **EQ-HWB- seeing** | How much difficulty did you have seeing? |
| --- | --- |
| **EQ-HWB- hearing** | How much difficulty did you have hearing? |
| **EQ-HWB-mobility** | How much difficulty did you have getting around inside and outside? |
| **EQ-HWB- activities** | How much difficulty did you have doing day-to-day activities? |
| **EQ-HWB-selfcare** | How much difficulty did you have washing, using the toilet, getting dressed, eating or caring for your appearance? |
| **EQ-HWB-sleep** | Have problems with your sleep? |
| **EQ-HWB-exhausted** | Feel exhausted? |
| **EQ-HWB-lonely** | Feel lonely? |
| **EQ-HWB-no support** | Feel that people did not support you? |
| **EQ-HWB-trouble remembering** | Have trouble remembering? |
| **EQ-HWB-trouble concentrating** | Have trouble concentrating or thinking clearly? |
| **EQ-HWB-anxious** | Feel anxious? |
| **EQ-HWB-unsafe** | Feel unsafe? (e.g. fear of falling, physical harm, abuse) |
| **EQ-HWB-frustrated** | Feel frustrated? |
| **EQ-HWB-sad or depressed** | Feel sad or depressed? |
| **EQ-HWB-nothing to look forward** | Feel you had nothing to look forward to? |
| **EQ-HWB-no control over your day-to-day life** | Feel you had no control over your day-to-day life? |
| **EQ-HWB-cope** | Feel unable to cope with day-to-day life? |
| **EQ-HWB-Accepted by others** | Did you feel accepted by others? |
| **EQ-HWB- feel good yourself** | Did you feel good about yourself? |
| **EQ-HWB-thing you want to do** | Could you do the things you wanted to do? |
| **EQ-HWB-physical pain** | Have physical pain? |
| **EQ-HWB-amount physical pain** | describe how much physical pain you had |
| **EQ-HWB-discomfort** | have physical discomfort? |
| **EQ-HWB-amount physical discomfort** | how much physical discomfort you had |
| **QOLACC-mobility** | I am able to get around as much as I want to |
| **QOLACC-pain management** | When I experience pain, it is well managed: |
| **QOLACC-emotional wellbeing** | I am generally happy: |
| **QOLACC-independence,** | I have as much independence as I want: |
| **QOLACC-social relationships** | I have good social relationships with family and friends: |
| **QOLACC-leisure activities/hobbies)** | I have leisure activities/ hobbies I enjoy: |
| **ASCOT-Control** | Which of the following statements best describes how much control you have over your daily life? By ‘control over daily life’ we mean having the choice to do things or have things done as you like... |
| **ASCOT-Cleanliness** | Thinking about keeping clean and presentable in appearance, which of the following statements best describes your situation? |
| **ASCOT-Food and drink** | Thinking about the food and drink you get, which of the following statements best describes your situation? |
| **ASCOT-Safety** | Which of the following statements best describes how safe you feel? By ‘feeling safe’ we mean how safe you feel both inside and outside the home. This includes fear of abuse, falling or other physical... |
| **ASCOT-Social participant and involvement** | Thinking about how much contact you have with people you like, which of the following statements best describes your social situation? |
| **ASCOT-Occupation** | Which of the following statements best describes how you spend your time? |
| **ASCOT-Accommodation** | Which of the following statements best describes how clean and comfortable your home is? |
| **ASCOT-Dignity** | Which of these statements best describes how the way you are helped and treated makes you think and feel about yourself? |
